# Oseltamivir in pregnancy and birth outcomes

**DOI:** 10.1186/s12879-018-3423-z

**Published:** 2018-10-16

**Authors:** Vera Ehrenstein, Nickolaj Risbo Kristensen, Brigitta Ursula Monz, Barry Clinch, Andy Kenwright, Henrik Toft Sørensen

**Affiliations:** 1 Department of Clinical Epidemiology, Aarhus University Hospital, Olof Palmes Allé 43-45, 8200 Aarhus N, Denmark; 2 F. Hoffmann-La Roche Ltd., Basel, Switzerland; 3 Roche Products Ltd., Welwyn Garden City, UK

**Keywords:** Congenital abnormalities, Epidemiology, H1N1 influenza, Oseltamivir

## Abstract

**Background:**

Prenatal exposure to influenza or fever is associated with risk of congenital malformations. Oseltamivir is used to treat influenza and to provide post-exposure prophylaxis. We examined the association between oseltamivir use during pregnancy and birth outcomes.

**Methods:**

This was a nationwide registry-based prevalence study with individual level data linkage, in a setting of universal health care access. We included all recorded pregnancies in Denmark in 2002–2013, and used data from population registries to examine associations between dispensings for oseltamivir during pregnancy (first trimester, second/third trimester, none) and congenital malformations, foetal death, preterm birth, foetal growth, and low 5-min Apgar score. Adjusted odds ratios (ORs) and 95% confidence intervals (CIs) were computed using propensity score matching.

**Results:**

The study included 946,176 pregnancies. Of these, 449 had first-trimester exposure and 1449 had second/third-trimester exposure to oseltamivir. Adjusted ORs following first-trimester exposure were 0.94 (95% CI 0.49 to 1.83) for any major congenital malformation and 1.75 (95% CI 0.51 to 5.98) for congenital heart defects, based on 7 exposed cases. The association with congenital heart defects was present for etiologically implausible exposure periods and for known safe exposures. There was no evidence of an association between prenatal exposure to oseltamivir and any of the other birth outcomes assessed.

**Conclusions:**

The study does not provide evidence of risk associated with oseltamivir treatment additional to that associated with influenza infection.

**Electronic supplementary material:**

The online version of this article (10.1186/s12879-018-3423-z) contains supplementary material, which is available to authorized users.

## Background

Influenza infection during first trimester of gestation is associated with a 2.0-fold increased risk of any major malformation; a 3.3-fold increased risk of neural tube defects; a 1.6-fold increased risk of congenital heart defects; and with increased risks of several other types of malformations [1, 2]. Congenital heart defects are common, affecting 5–11 of 1000 live births [3], underscoring the importance of treatment and prevention of first-trimester viral infections and their sequelae. During the 2009–2010 pandemic, H1N1 influenza A infection was associated with adverse pregnancy outcomes [4, 5], while treatment with a neuraminidase inhibitor (NAI) was associated with reduced risks of admission to intensive care units and lower mortality among pregnant women [4, 6]. Oseltamivir is a NAI used in treatment and post-exposure prophylaxis of influenza [7]. Evidence about pregnancy outcomes following oseltamivir exposure is reassuring [8–16], including a recent study based on routine health records from four European countries reporting no evidence of an increased risk of several birth outcomes following a NAI dispensing any time during gestation [16]. Nevertheless, previous studies had limitations, which include, potential selection bias from lack of data on abortuses, and potential misclassification of the outcome, which could dilute associations.

We examined safety of prenatal exposure to oseltamivir as measured by major congenital malformations, preterm birth, reduced foetal growth, low 5-min Apgar score, or foetal death. We addressed several limitations of previous studies by including pregnancies ending in abortive outcomes and ascertaining malformations through the first birthday; using a validated algorithm for congenital heart defects; and applying advanced methods of confounding control.

## Methods

### Data sources

We linked data from four population-based nationwide registries in Denmark: Danish Civil Registration System [17], Danish Medical Birth Registry [18], Danish National Patient Registry [19], and Danish National Prescription Registry [20]. Additional file 1: Table S1 provides a detailed description of all data sources, including specific types of data originating from each.

### Study design, population, and period

We included all pregnancies in Denmark that started and ended between 01 January 2002 and 31 December 2013. Pregnancies ending in a live birth or a stillbirth (≥22 gestational weeks) were identified in the Danish Medical Birth Registry. Pregnancies ending earlier than 22 gestational weeks in abortive outcomes were identified from hospital diagnoses recorded in the Danish National Patient Registry. Starting in 2007, the Danish National Patient Registry had information on congenital malformations identified during second-trimester therapeutic pregnancy terminations.

### Exposure

The Danish National Prescription Registry provided information on dispensings for oseltamivir at outpatient (community) pharmacies. The following mutually exclusive categories of oseltamivir exposure during pregnancy were defined: exposure during the first trimester regardless of exposure in the second or third trimester; exposure during the second or the third trimester but not in the first trimester; and no exposure at any time during pregnancy (the reference category). Because organogenesis is complete in the first trimester, we examined association of first-trimester oseltamivir exposure with congenital malformations. For the remaining birth outcomes, oseltamivir exposure at any trimester was considered.

### Outcomes

Congenital malformations, identified from diagnoses recorded at therapeutic second trimester abortions (2007–2013), at stillbirth, and up to 1 year postnatally in liveborn infants, were classified according to the major EUROCAT categories [3]. The Danish National Patient Registry is nearly 99% complete for diagnoses of congenital malformations [21]. For congenital heart defects, we used an algorithm developed specifically for the Danish National Patient Registry, based on the EUROCAT-specified diagnostic codes combined with therapeutic cardiac procedures [22]. The positive predictive value of this algorithm, estimated on a random sample of cases observed in this study, was 94.6% (95% confidence interval 89.2% to 97.7%). The other pregnancy outcomes were stillbirth at ≥22 weeks of gestation; foetal death (spontaneous or induced abortion before 22 weeks of gestation); preterm birth (gestational age 22- < 37 weeks) among live and stillbirths; small for gestational age (SGA) (birth weight below 10th percentile of the sex- and gestational-week-specific weight distribution) among live and stillbirths; and low 5-min Apgar score (< 7) among live births. For non-singleton pregnancies, a given outcome was considered present if recorded in at least one foetus/newborn.

### Covariates

We assessed the following covariates based on their known associations with the birth outcomes: maternal age at conception, calendar year of conception, smoking as reported at the first prenatal visit (for live and stillbirths); pre-pregnancy body mass index (BMI) for live and stillbirths; mode of delivery; parity; marital status; birth of a previous child with a malformation (since 1994); indicators of maternal health care utilization (hospitalizations, visits to hospital outpatient specialist clinics, emergency room visits, dispensings for specific drug classes); maternal inpatient or outpatient morbidity (respiratory disease, cardiovascular disease, haematological disease, diabetes, neurological disease, liver or kidney disease, rheumatic disease, inflammatory bowel disease, obesity, immunodeficiency, disorders of female pelvic organs/genital tract, hospital contact for injury or poisoning); maternal outpatient dispensings for antidepressants, antiepileptics, antidiabetics, antihypertensives, drugs for ulcer/gastroesophageal reflux, oral contraceptives, drugs for in-vitro fertilization, thyroid hormones, systemic corticosteroids, non-steroidal anti-inflammatory drugs, opiates, and systemic anti-infective agents other than oseltamivir. Data on all diagnoses originated from inpatient or outpatient hospital diagnoses (secondary care), while data on medication dispensings originated from primary care and outpatient prescribing. The covariates were ascertained during 12 months preconception. Information on influenza status was not available from any data source. Definitions of the study variables appear in Additional file 1: Table S2 and Table S3.

### Statistical analyses

We described the distributions of the pregnancy characteristics according to exposure to oseltamivir using appropriate descriptive statistics. For all outcomes except spontaneous or induced abortions, prevalence was used as the measure of occurrence. Crude and adjusted odds ratios (ORs) were computed using logistic regression. Pregnancies that ended before the second trimester were excluded from the analyses of second/third trimester exposure. For abortions, incidence rate was used, with hazard ratios estimated via Cox’s proportional-hazards regression, with oseltamivir exposure treated as a time-varying variable [23]. All estimates were reported with 95% confidence intervals (CIs).

Confounding was addressed using two approaches: propensity score matching and conventional adjustment using multivariate regression with generalised estimating equations to account for within-woman correlation. Propensity score matching was considered superior in control of measured confounding, while conventional adjustment allowed use of all available observations and provided the context to evaluate the direction of estimates’ change in response to tighter confounding control [24].

A propensity score for each pregnancy was computed, using logistic regression, as the probability of an oseltamivir dispensing given the covariates. Separate propensity scores were computed for the first-trimester and for the second/third-trimester exposure. Unexposed pregnancies were matched to exposed pregnancies on propensity score using nearest-neighbour matching with a caliper width of 0.2 standard deviations of the logit of the propensity score [25]. The balance of baseline characteristics was assessed post-matching, using standardised mean differences, whereby a value of ≤0.1 was considered indicative of balance. Per protocol, up to 100 oseltamivir-unexposed pregnancies were planned to be matched to each oseltamivir-exposed pregnancy. Post-matching assessment of the resulting balance indicated that only 1:1 matching achieved the target covariate balance. This 1:1 matched sample was used in propensity-score analysis, as it was deemed to remove most of the measured confounding. The covariates included in the propensity scores and the balancing statistics before and after matching are described in Additional file 1: Tables S4–S5, and Figure S1.

In conventionally adjusted analyses, we included all covariates with prevalence ≥5% or those inducing a >10% change in the crude OR. The final model included binary variables for parity (0 vs. > 0); marital status; smoking; obesity (BMI ≥30 kg/m^2^ or a hospital-based diagnosis of obesity); any chronic illness (cardiovascular disease, haematological disease, diabetes, neurological disease, liver or kidney disease, rheumatic disease or inflammatory bowel disease); and respiratory disease. In addition, all models included variables for mother’s age at conception (as a cubic spline) and for prior delivery of a child with a malformation. Smoking is not recorded for pregnancies ending in abortive outcomes; therefore the sensitivity analyses that contained such pregnancies were not adjusted for smoking.

The main analyses were conducted based on pregnancies ending in a live or stillbirth using propensity score to control for confounding. Since confounding by indication was expected to persist in this setting, several prespecified and post hoc sensitivity analyses were conducted for the malformation outcomes to obtain indirect evidence on confounding extent. First, we repeated the main analyses while including malformation diagnoses from terminated pregnancies (for pregnancies in 2007–2013). Second, we excluded mothers with a prior delivery of a child with a malformation. Third, we assessed risks of malformations associated with dispensing for oseltamivir during the main organogenesis period (gestational weeks 4–10 [26]). Fourth, we conducted several ‘negative control’ analyses [27]: examining effects of oseltamivir dispensing during periods etiologically implausible with respect to inducing major malformations (12 to 3 months preconception; second/third trimester of pregnancy). Fifth, we repeated the analysis replacing first-trimester exposure to oseltamivir with first-trimester exposure to penicillin, which is an anti-infective agent without evidence of teratogenicity [28] but presumed to correlate with presence of an infectious process, including fever. Finally, we examined the distribution of specific types of congenital heart defects for potential clustering, as clustering would support a causal association.

The analyses were conducted using SAS®, version 9.4 (Cary, NC, USA). Results were presented only when the individual cell counts in tables exceeded 5 observations, as specified by the Danish Data Protection Law (www.datatilsynet.dk) and/or regulations of Statistics Denmark (www.dst.dk).

## Results

**Fig. 1:**
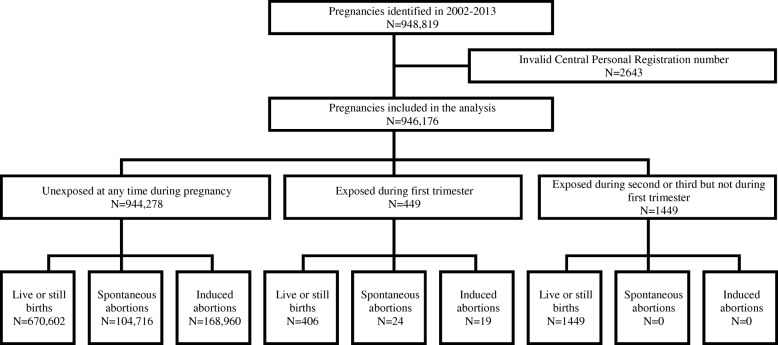
Identification of pregnancies beginning and ending in 2002–2013, Denmark

Between 01 January 2002 and 31 December 2013, 948,819 pregnancies started and ended in Denmark. After excluding 2643 (0.3%) pregnancies with invalid personal identifiers, 946,176 pregnancies remained in the analysis. Among these, 1898 (0.2%) pregnancies were exposed to oseltamivir: 449 during the first trimester and 1449 during the second or third trimester (Fig. 1). Of the oseltamivir-exposed pregnancies, 92% were exposed during 2009–2010. Table 1 presents characteristics of pregnancies included in the main analysis according to oseltamivir exposure, before and after propensity score matching.

**Table 1:** Characteristics of pregnancies resulting in a live or still birth, by exposure to oseltamivir

Characteristic	Before propensity score matching			After propensity score matching			
	Unexposed to oseltamivir during pregnancy	Exposed during first trimester	Exposed during second or third trimester	Unexposed to oseltamivir during pregnancy	Exposed during first trimester	Unexposed to oseltamivir during pregnancy	Exposed during second or third trimester
Number	670,602	406	1449	397	397	1420	1420
Age at conception (years)							
< 20	14,636 (2.2)	11 (2.7)	32 (2.2)	6 (1.5)	11 (2.8)	23 (1.6)	31 (2.2)
20-35	549,563 (82.0)	310 (76.4)	1114 (76.9)	301 (75.8)	303 (76.3)	1109 (78.1)	1088 (76.6)
≥ 35	106,403 (15.9)	85 (20.9)	303 (20.9)	90 (22.7)	83 (20.9)	288 (20.3)	301 (21.2)
Age at conception (years)							
Median (IQR)	30 (26–33)	30 (27–34)	30 (27–34)	31 (27–34)	30 (27–34)	31 (27–34)	30 (27–34)
Mean (SD)	30.0 (4.9)	30.7 (5.0)	30.8 (4.9)	31.1 (4.9)	30.8 (5.0)	31.0 (4.8)	30.8 (4.9)
Calendar year of conception							
2002-2008	426,697 (63.6)	18 (4.4)	60 (4.1)	241 (60.7)	18 (4.5)	845 (59.5)	60 (4.2)
2009-2010	118,496 (17.7)	376 (92.6)	1335 (92.1)	78 (19.6)	367 (92.4)	265 (18.7)	1310 (92.3)
2011-2013	125,409 (18.7)	12 (3.0)	54 (3.7)	78 (19.6)	12 (3.0)	310 (21.8)	50 (3.5)
Unmarried	357,106 (53.3)	191 (47.0)	663 (45.8)	184 (46.3)	185 (46.6)	623 (43.9)	645 (45.4)
Pre-pregnancy body mass index (kg/m^2^)							
< 18.5	24,336 (3.6)	20 (4.9)	46 (3.2)	6 (1.5)	20 (5.0)	55 (3.9)	46 (3.2)
18.5- < 25.0	355,498 (53.0)	238 (58.6)	835 (57.6)	204 (51.4)	233 (58.7)	766 (53.9)	822 (57.9)
25.0- < 30.0	118,521 (17.7)	84 (20.7)	305 (21.0)	75 (18.9)	83 (20.9)	269 (18.9)	300 (21.1)
≥ 30	70,259 (10.5)	54 (13.3)	212 (14.6)	48 (12.1)	52 (13.1)	166 (11.7)	206 (14.5)
Missing	101,988 (15.2)	10 (2.5)	51 (3.5)	64 (16.1)	9 (2.3)	164 (11.5)	46 (3.2)
Smoking							
No	561,830 (83.8)	351 (86.5)	1258 (86.8)	358 (90.2)	351 (88.4)	1275 (89.8)	1258 (88.6)
Yes	95,528 (14.2)	46 (11.3)	162 (11.2)	39 (9.8)	46 (11.6)	145 (10.2)	162 (11.4)
Missing^a^	13,244 (2.0)	9 (2.2)	29 (2.0)	–	–	–	–
Parity							
0	314,229 (46.9)	136 (33.5)	441 (30.4)	122 (30.7)	132 (33.2)	415 (29.2)	427 (30.1)
1	242,531 (36.2)	183 (45.1)	637 (44.0)	187 (47.1)	179 (45.1)	654 (46.1)	627 (44.2)
2	86,863 (13.0)	64 (15.8)	296 (20.4)	67 (16.9)	63 (15.9)	270 (19.0)	292 (20.6)
> 2	26,979 (4.0)	23 (5.7)	75 (5.2)	21 (5.3)	23 (5.8)	81 (5.7)	74 (5.2)
Prior delivery of a child with a malformation (since 1994)	20,171 (3.0)	16 (3.9)	67 (4.6)	19 (4.8)	16 (4.0)	72 (5.1)	64 (4.5)
Hospital history during 12 months before conception^b^							
At least one inpatient hospitalization	124,627 (18.6)	83 (20.4)	334 (23.1)	75 (18.9)	79 (19.9)	270 (19.0)	327 (23.0)
At least one visit to outpatient specialist clinic	232,129 (34.6)	169 (41.6)	609 (42.0)	155 (39.0)	165 (41.6)	570 (40.1)	597 (42.0)
At least one emergency room visit	85,760 (12.8)	80 (19.7)	215 (14.8)	69 (17.4)	77 (19.4)	180 (12.7)	211 (14.9)
Respiratory diseases	33,405 (5.0)	39 (9.6)	90 (6.2)	38 (9.6)	39 (9.8)	89 (6.3)	89 (6.3)
Cardiovascular disease	7760 (1.2)	7 (1.7)	30 (2.1)	5 (1.3)	6 (1.5)	15 (1.1)	29 (2.0)
Haematological disease	2364 (0.4)	5 (1.2)	15 (1.0)	0 (0.0)	5 (1.3)	8 (0.6)	15 (1.1)
Diabetes	10,590 (1.6)	5 (1.2)	38 (2.6)	7 (1.8)	5 (1.3)	29 (2.0)	37 (2.6)
Neurological disease^b^	5654 (0.8)	5 (1.2)	20 (1.4)	–	–	–	–
Obesity^c^	73,216 (10.9)	56 (13.8)	222 (15.3)	49 (12.3)	54 (13.6)	174 (12.3)	216 (15.2)
Disorders of female pelvic organs/genital tract	44,730 (6.7)	32 (7.9)	86 (5.9)	25 (6.3)	32 (8.1)	70 (4.9)	84 (5.9)
Hospital contact for injury or poisoning	60,037 (9.0)	51 (12.6)	148 (10.2)	51 (12.8)	50 (12.6)	130 (9.2)	144 (10.1)
Use of prescription medication in the 12 months before conception							
Antidepressants	34,293 (5.1)	30 (7.4)	115 (7.9)	29 (7.3)	29 (7.3)	105 (7.4)	109 (7.7)
Drugs for ulcer/gastroesophageal reflux	26,456 (3.9)	27 (6.7)	84 (5.8)	18 (4.5)	26 (6.5)	80 (5.6)	83 (5.8)
Oral contraceptives	243,936 (36.4)	156 (38.4)	534 (36.9)	173 (43.6)	153 (38.5)	517 (36.4)	523 (36.8)
Drugs for in-vitro fertilization	54,563 (8.1)	43 (10.6)	116 (8.0)	35 (8.8)	43 (10.8)	103 (7.3)	114 (8.0)
Thyroid hormones	7284 (1.1)	8 (2.0)	25 (1.7)	8 (2.0)	8 (2.0)	31 (2.2)	24 (1.7)
Systemic corticosteroids	13,005 (1.9)	17 (4.2)	49 (3.4)	17 (4.3)	16 (4.0)	41 (2.9)	49 (3.5)
Non-steroidal anti-inflammatory drugs	98,907 (14.7)	78 (19.2)	268 (18.5)	66 (16.6)	77 (19.4)	252 (17.7)	261 (18.4)
Opiates	21,777 (3.2)	18 (4.4)	58 (4.0)	17 (4.3)	17 (4.3)	76 (5.4)	57 (4.0)
Systemic anti-infective agents other than oseltamivir	278,680 (41.6)	195 (48.0)	734 (50.7)	189 (47.6)	190 (47.9)	740 (52.1)	719 (50.6)
Number of different drugs classes dispensed, median (IQR)	1 (1–2)	2 (1–3)	2 (1-3)	2 (1–3)	2 (1–3)	2 (1–3)	2 (1–3)

*IQR* interquartile range, *SD* standard deviation

^a^Matching of the unexposed pregnancies was done separately for those exposed in the first trimester and those exposed in the second or third trimester. No matching of pregnancies with missing data on smoking

^b^Not reported for the following protocol-specified characteristics because of low (< 5) group counts: liver and kidney disease; rheumatic disease; inflammatory bowel disease; immunodeficiency, and use of antiepileptics. In the propensity-score matched dataset neurologic diseases also not reported. ^c^Defined by pre-pregnancy body mass index or a hospital diagnosis of obesity. Data are n (%) unless otherwise specified

Table 2 shows crude and adjusted odds ratios for the association of first-trimester exposure to oseltamivir with congenital malformations. Of the 19 first-trimester-exposed pregnancies with major congenital malformations, 8 were congenital heart defects. Among the 670,602 oseltamivir-unexposed pregnancies, prevalence of any major malformation was 3.7% and prevalence of congenital heart defects was 0.7%. Among the 406 pregnancies with first-trimester oseltamivir exposure, the prevalence of any malformation was 4.7% and the prevalence of congenital heart defects was 2.0%. The odds ratios from propensity-score matched regression analysis were 0.94 (95% CI 0.49 to 1.83) for any malformation and 1.75 (95% CI 0.51 to 5.98) for congenital heart defects. Associations for congenital heart defects were also observed in the negative control sensitivity analyses of dispensing during second or third trimester (Table 3).

**Table 2:** First-trimester exposure to oseltamivir and congenital malformations among live or stillbirths

Outcome	Unexposed *N* = 670,602	Exposed *N* = 406	Crude odds ratio (95% CI)	Adjusted odds ratio (95% CI)	
				Conventional regression analysis^a^	Propensity-score matched regression analysis^b^
Any major congenital malformation	24,773 (3.7%)	19 (4.7%)	1.28 (0.81 to 2.03)	1.25 (0.78 to 2.01)	0.94 (0.49 to 1.83)
Congenital heart defects	4795 (0.7%)	8 (2.0%)	2.79 (1.39 to 5.62)	2.51 (1.19 to 5.31)	1.75 (0.51 to 5.98)

*CI* confidence interval

^a^Adjusted for age at conception, parity, smoking, marital status, obesity, prior delivery of a child with a malformation, respiratory diseases, any other chronic illness (cardiovascular disease, haematological disease, diabetes, neurological disease [including antidepressants or antiepileptics use], liver or kidney disease, rheumatic disease or inflammatory bowel disease) during the 12 months before conception

^b^Sample size: exposed = 397; unexposed = 397; prevalence of any major malformation, exposed/unexposed: 4.5%/4.8%; prevalence of congenital heart defects, exposed/unexposed: 1.8%/1.0%

**Table 3:** Sensitivity analyses of the outcome of congenital malformations following exposure to oseltamivir in pregnancy

	Conventional regression analysis			Propensity-score matched regression analysis		
	Unexposed	Exposed	Odds ratio (95% CI) ^a^	Unexposed	Exposed	Odds ratio (95% CI)^b^
Analysis including pregnancies terminated due to congenital malformations (2007-2013), prespecified						
Number	521,037	432		432	432	
Any major congenital malformation	16,692 (3.2%)	22 (5.1%)	1.58 (1.03 to 2.44)	20 (4.6%)	22 (5.1%)	1.10 (0.60 to 2.02)
Congenital heart defects	3005 (0.6%)	8 (1.9%)	3.17 (1.57 to 6.40)	4 (0.9%)	8 (1.9%)	2.00 (0.60 to 6.64)
Pregnancies among women without an earlier pregnancy resulting in a malformed child*,* post hoc						
Number	650,431	390		381	381	
Any major malformation	23,712 (3.7%)	18 (4.6%)	1.26 (0.77 to 2.04)	20 (5.3%)	17 (4.5%)	0.84 (0.43 to 1.64)
Congenital heart defects	4598 (0.7%)	8 (2.1%)	2.68 (1.27 to 5.68)	5 (1.3%)	7 (1.8%)	1.50 (0.42 to 5.32)
First trimester exposure to oseltamivir, defined as oseltamivir dispensing during 4-10 weeks of gestation, post hoc						
Number	670,602	212		205	205	
Any major malformation	24,773 (3.7%)	8 (3.8%)	0.91 (0.43 to 1.95)	8 (3.9%)	7 (3.4%)	0.88 (0.32 to 2.41)
Congenital heart defects	4795 (0.7%)	5 (2.4%)	2.70 (1.00 to 7.29)	N/A		
Oseltamivir dispensing in second or third trimester (post-organogenesis), prespecified						
Number	670,602	1449		1420	1420	
Any major malformation	24,773 (3.7%)	64 (4.4%)	1.18 (0.91 to 1.53)	51 (3.6%)	61 (4.3%)	1.21 (0.82 to 1.77)
Congenital heart defects	4795 (0.7%)	21 (1.5%)	2.10 (1.36 to 3.24)	6 (0.4%)	21 (1.5%)	3.50 (1.41 to 8.67)
Exposure to oseltamivir between 12 and 3 months preconception, post hoc						
Number	669,934	538		530	530	
Any major malformation	24,745 (3.7%)	24 (4.5%)	1.20 (0.80 to 1.80)	19 (3.6%)	24 (4.5%)	1.28 (0.69 to 2.37)
Congenital heart defects	N/A					
First-trimester exposure to penicillin, *post hoc*^c^						
Number	471,117	70,309		Analysis not conducted		
Any major malformation	17,148 (3.6%)	2798 (4.0%)	1.09 (1.04 to 1.13)			
Congenital heart defects	3296 (0.7%)	599 (0.9%)	1.17 (1.07 to 1.29)			

*CI* confidence interval, *N/A* not applicable (counts too low to report)

^a^Adjusted for age at conception, parity, smoking, marital status, obesity, prior delivery of a child with a malformation, respiratory diseases, any other chronic illness (cardiovascular disease, haematological disease, diabetes, neurological disease [including use of antidepressants or antiepileptics], liver or kidney disease, rheumatic disease, or inflammatory bowel disease) during 12 months before conception

^b^Variables included in estimation of propensity scores are listed in the Additional file 1

^c^Exposure to penicillin for malformations (any, congenital heart defects) among live born and stillborn infants conceived and delivered in 2002-2013 in Denmark

Table 4 presents results for the outcomes SGA, preterm birth, low 5-min Apgar score, and stillbirth. Based on propensity-score adjusted analyses, most odds ratios were close to 1.0. Some propensity-score-matched estimates were imprecise and therefore should be interpreted with caution. Table 5 shows the association between pregnancy exposure to oseltamivir and spontaneous or induced abortions, with oseltamivir as a time-varying exposure, based on 861 pregnancies exposed to oseltamivir. Adjusted incidence rate ratios associated with oseltamivir exposure were 0.99 (95% CI 0.66 to 1.48) for spontaneous abortion and 0.64 (95% CI 0.41 to 1.00) for induced abortion. No clustering of specific congenital heart defects was observed (data not shown).

**Table 4:** Prenatal exposure to oseltamivir and SGA, preterm birth, low 5-min Apgar score, and stillbirth

	Unexposed	Exposed	Crude odds ratio (95% CI)	Conventional regression analysis, odds ratio (95% CI)	Propensity score-matched regression analysis, odds ratio (95% CI)^c^
First trimester					
Number	670,602	406			
Small for gestational age	64,944 (9.7%)	32 (7.9%)	0.80 (0.56 to 1.14)	0.88 (0.61 to 1.26)	0.75 (0.46 to 1.24)
Preterm birth	42,399 (6.3%)	28 (6.9%)	1.10 (0.75 to 1.61)	1.21 (0.83 to 1.79)	0.87 (0.52 to 1.46)
Low 5-min Apgar score^a^	5429 (0.8%)	5 (1.2%)	1.52 (0.63 to 3.68)	1.64 (0.68 to 3.97)	1.00 (0.29 to 3.45)
Second/third trimester					
Number	670,602	1449			
Small for gestational age	64,944 (9.7%)	121 (8.3%)	0.85 (0.71 to 1.02)	0.94 (0.77 to 1.13)	0.84 (0.64 to 1.10)
Preterm birth	42,399 (6.3%)	79 (5.4%)	0.85 (0.68 to 1.07)	0.89 (0.70 to 1.12)	0.85 (0.62 to 1.16)
Low 5-min Apgar score^a^	5429 (0.8%)	10 (0.7%)	0.85 (0.46 to 1.59)	0.92 (0.49 to 1.71)	1.25 (0.49 to 3.17)
Stillbirth^b^	3047 (0.4%)	7 (0.5%)	1.06 (0.51 to 2.24)	1.13 (0.50 to 2.52)	1.20 (0.37 to 3.93)

Data are n (%) or odds ratio (95% CI) unless otherwise specified

*SGA* small for gestational age, *CI* confidence interval

^a^Live born only

^b^Live or stillborn at ≥22 weeks of gestation; data not shown for first-trimester exposure because of low counts

^c^Variables included in estimation of propensity scores are listed in the Additional file 1

**Table 5:** Exposure to oseltamivir in pregnancy and spontaneous or induced abortions

Type of abortion	Exposed^a^		Unexposed		Crude hazard ratio (95% CI)	Adjusted^b^ hazard ratio (95% CI)
Spontaneous or induced	43	122	273,676	293,663	0.77 (0.57 to 1.04)	0.79 (0.59 to 1.07)
Spontaneous	24	122	104,716	293,663	1.00 (0.67 to 1.50)	0.99 (0.66 to 1.48)
Induced	19	122	168,960	293,663	0.59 (0.38 to 0.93)	0.64 (0.41 to 1.00)

*CI* confidence interval

^a^Based on 861 gestations exposed to oseltamivir

^b^Adjusted for age at conception, parity, smoking, marital status, obesity, prior delivery of a child with a malformation, respiratory diseases, any other chronic illness [cardiovascular disease, haematological disease, diabetes, neurological disease (including use of antidepressants or antiepileptics), liver or kidney disease, rheumatic disease or inflammatory bowel disease] during 12 months before conception

^c^Exposure to oseltamivir is analysed as time-varying variable

## Discussion

### Main findings

In this population-based study, prenatal exposure to oseltamivir was not associated with increased risks of any major congenital malformation, foetal death, preterm birth, SGA or low 5-min Apgar score. For congenital heart defects, defined using a validated algorithm with high positive predictive value and completeness, exposure to oseltamivir during the first trimester was associated with an adjusted odds ratio of 1.75 (95% CI 0.51 to 5.98) based on live and stillbirths, and with an adjusted odds ratio of 2.00 (95% CI 0.60 to 6.64) after inclusion of malformations from terminated pregnancies. The association persisted for oseltamivir exposure in the second or third trimester, i.e., after completion of the organogenesis. There was no clustering of specific congenital heart defects among foetuses with first-trimester oseltamivir exposure. Because of low prevalence of oseltamivir exposure, associations with other major congenital malformations could not be evaluated.

### Limitations

Important limitations of the present analysis are the low number of exposed cases and the lack of systematic data on influenza status. It is plausible to assume that during the 2009–2010 H1N1 influenza A pandemic, most oseltamivir use in pregnancy was therapeutic rather than prophylactic. This essentially guaranteed confounding by indication, especially since the unexposed pregnancies, the overwhelming majority of which were not affected by influenza, were used as the comparator in the analysis. An ideal comparator population would be composed of pregnancies affected by influenza but not treated with oseltamivir to provide the background risk of outcomes in influenza affected population. Instead, the comparator population of unexposed pregnancies represented the prevalence of congenital malformations in the general Danish population, i.e., an overall prevalence of malformations close to that reported for Denmark by the EUROCAT, based on a representative sample (2002–2012 total prevalence per 1000 births: 30 [95% CI 28 to 31] for any malformation; 9.1 [95% CI 8.4 to 10.0] for congenital heart defects [3]). The most recent analysis involving Scandinavian data [16] excluded pregnancies with a hospital-based diagnosis of influenza. This was done to reduce confounding by indication, but may have potentially introduced selection bias by excluding the most severely affected pregnancies from the study population. In our study, excluding women with a hospital diagnosis of influenza did not materially affect the findings (conventionally adjusted OR for any major congenital malformation 1.20 [95% CI: 0.74 to 1.95]).

Several considerations point to residual confounding These include increasing attenuation of odds ratios in response to closer confounding control; persisting associations for the negative control exposures, for which no association was expected unless caused by confounding – i.e., in periods after the organogenesis is expected to be complete. At the same time, the ORs for congenital heart defects were similar to those reported in other studies for first-trimester fever (1.54 [95% CI 1.37 to 1.74]) [29] or first-trimester influenza infection (1.56 [95% CI 1.13 to 2.14]) [1]. The lower precision of the odds ratios obtained in the propensity score matched analysis was the trade-off taken for maximising validity via 1:1 matching. Other limitations of this analysis are potential misclassification of exposure status by relying on dispensing records, by lack of information on oseltamivir dispensed during hospital stays, and by potential errors in recorded gestational age.

### Other evidence

Taken together, the available evidence is not consistent with harmful pregnancy effects of oseltamivir or other NAIs [8, 10–16]. A Canadian study of more than 55,000 pregnant women, including 1237 exposed to oseltamivir during the H1N1 pandemic, reported no evidence of an association between prenatal exposure to oseltamivir and preterm birth, low Apgar scores, or poor foetal growth [12]. Similarly, a study in Texas based on 135 oseltamivir-exposed pregnancies did not suggest harmful pregnancy effects [13]. The most recent study, conducted by Graner et al. [16], used linked databases from four European countries, including the same source data for Denmark as used in the current study and investigated similar outcomes. Graner et al. identified seven cases of congenital heart malformations in 814 first trimester oseltamivir-exposed pregnancies, resulting in a (conventionally) adjusted odds ratio of 0.96 (95% CI 0.43 to 2.15). Our study detected eight congenital heart defects in less than half as many exposed pregnancies (*N* = 406). This may be explained by the extension of the case detection period to up to 1 year postnatally. The differences in the conventionally adjusted odds ratios in our study compared with the study by Graner et al. (2.51 versus 0.96) may have resulted from our decision not to exclude pregnancies with hospital influenza diagnoses, combined with the higher prevalence of congenital heart defects among offspring of exposed women in our study population.

### Interpretation

The OR for congenital heart defects associated with the first-trimester exposure to oseltamivir was of comparable size to that reported for influenza infection or fever, indicating that despite close control of confounding, in the setting of pregnancy, a nearly full confounding by influenza status is likely. The association was also observed during etiologically implausible periods, such as periods after completion of organogenesis; furthermore, the ORs weakened in response to successive control of measured confounding, from crude ORs (fully confounded) to conventionally adjusted ORs (some residual confounding) to propensity-score adjusted ORs (least residual confounding). Lack of clustering of specific congenital heart defects among foetuses with first-trimester oseltamivir exposure although does not disprove it, argues against the causality underlying the observed association [30]. Thus, this study in the context of the available evidence is consistent with adverse pregnancy outcomes being associated with influenza infection itself.

## Conclusions

The study does not provide evidence of risk associated with oseltamivir treatment additional to that previously known to be associated with influenza infection.

## Additional file

Additional file 1: Online supplement to Ehrenstein et al. Oseltamivir in pregnancy and birth outcomes. **Table S1.** Danish registries used to assemble the analysis dataset. **Table S2.** ATC, ICD, and procedure codes used to identify study variables other than congenital malformations. **Table S3.** EUROCAT algorithms to identify major congenital malformations. **Table S4.** Types of propensity scores and analysis sets. **Table S5.** Post-matching mean differences in variables included in propensity score estimation. **Figure S1.** Distribution of propensity score in the matched sample for first-trimester (A) or second/third trimester (B) exposure to oseltamivir and the 1:1 matched unexposed pregnancies. (DOCX 217 kb)

## Abbreviations

BMI: Body mass index
CI: Confidence interval
ICD-10: International Classification of Diseases, Tenth Revision
IQR: Interquartile range
N/A: Not applicable
NAI: Neuraminidase inhibitor
OR: Odds ratio
SD: Standard deviation
SGA: Small for gestational age
