# Supplementary material for: Oseltamivir in pregnancy and birth outcomes
Source: BMC Infect Dis. 2018 Oct 16;18:519. doi: 10.1186/s12879-018-3423-z (PMC6192366; doi:10.1186/s12879-018-3423-z)
Supplement: Supplementary file 1 — Online supplement to Ehrenstein et al. Oseltamivir in pregnancy and birth outcomes. Table S1. Danish registries used to assemble the analysis dataset. Table S2. ATC, ICD, and procedure codes used to identify study variables other than congenital malformations. Table S3. EUROCAT algorithms to identify major congenital malformations. Table S4. Types of propensity scores and analysis sets. Table S5. Post-matching mean differences in variables included in propensity score estimation. Figure S1. Distribution of propensity score in the matched sample for first-trimester (A) or second/third trimester (B) exposure to oseltamivir and the 1:1 matched unexposed pregnancies. (DOCX 217 kb) [file 12879_2018_3423_MOESM1_ESM.docx]

**Additional file 1**

**Online supplement to Ehrenstein et al. Oseltamivir in pregnancy and birth outcomes**

| **Table S1. Danish registries used to assemble the analysis dataset** | |
| --- | --- |
| Data source (year established) | Type of data and brief description of data source |
| Danish Civil Registration System (1968-) | Source of data for: personal identifier for mothers and infants, vital status, migrations, marital status  *The Danish Civil Registration System* assigns a unique identifier to all persons who are born in or immigrate to Denmark (the central personal [CPR] number) since 1968. The CPR number, which encodes sex and date of birth, was used to link data from all registries on individual (pregnancy) level. |
| Danish Medical Birth Registry (1973-) | Source of data for: gestational age, birth weight, parity (defined here as number of previous live births), 5-minute Apgar score, live births, stillbirths, smoking during pregnancy, body mass index (BMI), date of conception (estimated from gestational age and date of birth).  *The Danish Medical Birth Registry* records all deliveries in Denmark from the last day of gestational week 22 onwards. |
| Danish National Patient Registry (1977-) | Source of data for: congenital malformations, spontaneous and induced abortions, maternal morbidity.  *The Danish National Patient Registry* captures inpatient stays, emergency-room visits, and visits at hospital-based specialist outpatient clinics at all Danish somatic hospitals since 1977. The registry tracks discharge diagnoses with dates, and information about procedures, with dates. The International Classification of Diseases, Tenth Revision (ICD-10) was used to code the diagnoses during the study period. |
| Danish National Prescription Registry (1995-) | Source of data for: outpatient dispensations of prescription medications  *The Danish National Prescription Registry* tracks all prescription dispensed in outpatient pharmacies in Denmark since 1995. Recorded information include data of dispensation, active substance coded using the Anatomical Therapeutic Chemical (ATC) code, amount sold, package size, and route of administration. |

| Table S2. ATC, ICD, and procedure codes used to identify study variables other than congenital malformations | | |
| --- | --- | --- |
| **Variable** | **Algorithm** |  |
| Oseltamivir dispensation | ATC: J05AH02 |  |
| Characteristics of the pregnant woman |  |  |
| Age at conception | Coded in the unique identifier |  |
| Smoking | Variable in the Medical Birth Registry (based on self-report at a prenatal visit) |  |
| Pre-gravid body mass index | Variable in the Medical Birth Registry |  |
| Parity | Variable in the Medical Birth Registry/Civil Registration System |  |
| Marital (‘civil’) status | Variable in the Medical Birth Registry/Civil Registration System |  |
| Mode of delivery | Variable in the Medical Birth Registry |  |
| History of giving birth with a child with malformation | From Medical Birth Registry |  |
| Number of distinct drug classes | Count of medications in the main (anatomic) ATC groups from the Danish National Prescription Registry |  |
| Number of inpatient hospitalizations | Count from the Danish National Patient Registry |  |
| Number of visits to hospital specialist outpatient clinics | Count from the Danish National Patient Registry |  |
| Number of emergency room visits | Count from the Danish National Patient Registry |  |
| Acute and chronic morbidity of pregnant women |  |  |
| Hospital diagnoses of influenza | ICD-10 Influenza: G051F, G051O, H671B, J09, J091, J091A, J091B, J099, J10, J100, J101, J101A, J101B, J101C, J108, J108A, J108B, J108C, J11, J110, J111, J111A, J111B, J111C, J118, J118A, J118B, J118C, I411A |  |
| Cardiovascular diseases | ICD10: I00-I79, Q20-Q28, ATC: C01-C03 |  |
| Haematological diseases | ICD10: D50-D84, D89, I80-I82 |  |
| Diabetes | ICD10: E10-E15, ATC: A10 |  |
| Neurological diseases | ICD10: G10-G99 |  |
| Liver and kidney disease | ICD10: K70-K79, N00-N19, N25-N28, Q61 |  |
| Rheumatic disease | ICD10: M05-M14, M30-M36 |  |
| Inflammatory bowel disease | ICD10: K50-K51, K523, K528, K529, ATC: A07E |  |
| Obesity | E65-E66, E68, pregravid BMI≥30 kg/m^2^ |  |
| Immunodeficiency or use of immunosuppressants | ICD10: D80-D84, D89, ATC: L04A |  |
| Disorders of female pelvic organs/genital tract | ICD10: N70-N98 |  |
| Hospital contact for injury or poisoning | ICD10: S00-T98 |  |
| Use of prescription medications |  |  |
| Antidepressants | ATC: N06A |  |
| Antiepileptics | ATC: N03A |  |
| Drugs for peptic ulcer/gastroesophageal reflux | ATC: A02B |  |
| Oral contraceptives | ATC: G03A |  |
| Drugs for in-vitro fertilization | ATC: G03G, G03DA04, H01AA, H01CC, L02AE01 |  |
| Thyroid hormones | ATC: H03AA |  |
| Systemic corticosteroids | ATC: H02A |  |
| Non-steroidal anti-inflammatory drugs (NSAIDs) | ATC: M01A, N02BA |  |
| Opiates | ATC: N02A |  |
| Systemic antiinfective agents | ATC: J (including antivirals other than oseltamivir) |  |
| Spontaneous abortion | ICD10: O021, O03 |  |
| Induced abortion | ICD10: O04-O06 |  |

| Table S3. EUROCAT algorithms to identify major congenital malformations | | | |
| --- | --- | --- | --- |
| EUROCAT Subgroups | ICD-10 | Comments | Excluded minor anomalies post-2005 |
| All anomalies/Any malformation* | Q-chapter, D215, D821, D1810, P350, P351, P371 |  | Exclude all minor anomalies as specified in Guide 1.4, section 3.2  Q101, Q102, Q103, Q105, Q135, Q170-Q175, Q179-Q182, Q184-Q187, Q189, Q211C, Q250 if GA <37 weeks, Q254E, Q256 if GA<37 weeks, Q261, Q270, Q314, Q315, Q320, Q331, Q381, Q382, Q385B, Q400, Q401, Q430, Q432, Q523, Q525, Q527, Q53, Q552B, Q552F, Q610, Q627, Q633, Q653‐Q656, Q662-Q678, Q680, Q682A, Q683-Q685, Q740G, Q752, Q753, Q760, Q764L, Q765, Q766A, Q766C, Q767C, Q825, Q833, Q845, Q899, Q950, Q951 |
| Nervous system | Q00, Q01, Q02, Q03, Q04, Q05, Q06, Q07 |  | Q0461, Q0782 (Crocodile tears, no Danish code) |
| Ear, face and neck | Q16, Q17, Q18 |  | Q170-Q175, Q179, Q180- Q182, Q184- Q187, Q1880 (Synophrys no Danish code), Q189 |
| Congenital Heart Defects (abortive outcomes and still births) | Q20-Q26 |  | Q211C, Q254E, Q261 |
| Congenital Heart Defects (live births) | Q20-Q26 | Exclude PDA with GA <37 weeks Exclude peripheral pulmonary artery stenosis with GA < 37 weeks  Therapeutic cardiac procedures as defined in ^1^ KFNG02, KFNG03, KFNG04, KFNG05, KFNA, KFNB, KFNC | Q211C, Q250 if GA <37 weeks and live birth,  Q254E (Persistent right aortic arch),  Q256 if GA<37 weeks,  Q261 |
| Respiratory | Q300, Q32- Q34 | Exclude Q336 | Q314, Q315, Q320, Q331 |
| Oro-facial clefts | Q35-Q37 | Exclude association with holoprosencephaly or anencephaly subgroups  Do not count in this category if these codes are present: Q00, Q041, Q042 |  |
| Digestive system | Q38-Q45, Q790 |  | Q381, Q382, DQ385B, Q400, Q401, Q4021 (no Danish code), Q430, Q432 |
| Abdominal wall defects | Q792, Q793, Q795 |  |  |
| Urinary | Q60-Q64, Q794 |  | Q610, Q627, Q633 |
| Genital | Q50-Q52, Q54-Q56 |  | Q523, Q525, Q527, Q552F, Q552B |
| Limb | Q65-Q74 |  | Q653-Q656, Q662-Q669, Q670-Q678, Q680, Q6810 (no Danish code), Q682A, Q683- Q685, Q740G |
| Other anomalies / syndromes |  |  |  |
| Chromosomal | Q90-Q92, Q93 , Q96- Q99 | Exclude microdeletions Q936 |  |
| Cases with more than one anomaly are only counted once in the “All Anomalies/Any malformation” subgroup.  (Source: <http://www.eurocat-network.eu/content/EUROCAT-Guide-1.4-Section-3.3.pdf>, adapted to present project, e.g., deleted references to non-ICD-10 classifications), adapted to the Danish ICD-10 coding. | | | |

| **Table S4. Types of propensity scores and analysis sets** | | |
| --- | --- | --- |
| Analysis set | Exposure to oseltamivir modelled | Variables included in the propensity score model |
| Live births, stillbirths, 2002-2013 | First-trimester | Age at conception (as a cubic spline), parity, history of giving birth to a child with a malformation, smoking, marital status, number of prescription groups besides antiinfective agents (≤2, >2), inpatient visits, outpatient visits, emergency room visits, respiratory diseases, obesity (based on BMI or hospital diagnoses), disorders of female pelvic organs/genital tract, antidepressants, oral contraceptives, drugs for in-vitro fertilization, NSAIDs, systemic antiinfective agents other than oseltamivir, any other prescription or morbidity except for injury/poisoning (because of collinearity with emergency room visits) |
|  | Second- or third-trimester |  |
| Live births, stillbirths, abortuses, 2007-2013 | First-trimester | All of the above except smoking; obesity modelled based on hospital diagnoses. |
|  | Second- or third-trimester |  |

Abbreviations: BMI: Body Mass Index, NSAID: Nonsteroidal anti-inflammatory drug

Table S5. Post-matching mean differences in variables included in propensity score estimation

|  | **Standardised mean difference after matching** | |
| --- | --- | --- |
| **Variable included in propensity score model** | **First trimester exposure to oseltamivir** | **Second or third trimester exposure to oseltamivir** |
| Age at conception (cubic spline) | 0·07 | 0·04 |
| Parity (0, >0) | 0·05 | 0·02 |
| History of giving birth to a child with a malformation | 0·04 | 0·03 |
| Smoking | 0·06 | 0·04 |
| Marital status | 0·01 | 0·03 |
| Respiratory diseases | 0·01 | 0·00 |
| Obesity | 0·04 | 0·09 |
| Disorders of female pelvic organs/genital tract | 0·07 | 0·04 |
| Any other chronic illnesses or prescription | 0·06 | 0·01 |
| At least one inpatient hospitalization | 0·03 | 0·10 |
| At least one visit to outpatient specialist clinic | 0·05 | 0·04 |
| At least one emergency room visit | 0·05 | 0·06 |
| Antidepressants | 0·00 | 0·01 |
| Oral contraceptives | 0·10 | 0·01 |
| Drugs for in-vitro fertilization | 0·07 | 0·03 |
| Non-steroidal anti-inflammatory drugs | 0·07 | 0·02 |
| Systemic antiinfective agents | 0·01 | 0·03 |
| Number of prescription groups (<=2, >2) | 0·01 | 0·00 |

Figure S1. Distribution of propensity score in the matched sample for first-trimester (A) or second/third trimester (B) exposure to oseltamivir and the 1:1 matched unexposed pregnancies

**A**

**B**
